# Disease-related gene module detection based on a multi-label propagation clustering algorithm

**DOI:** 10.1371/journal.pone.0178006

**Published:** 2017-05-19

**Authors:** Xue Jiang, Han Zhang, Xiongwen Quan, Zhandong Liu, Yanbin Yin

**Affiliations:** 1 College of Computer and Control Engineering, Nankai University, Tianjin 300350, China; 2 Tianjin Key Laboratory of Intelligent Robotics, Nankai University, Tianjin 300350, China; 3 Department of Pediatrics-Neurology, Baylor College of Medicine, Houston, TX 77030, United States of America; 4 Department of Biological Sciences, Northern Illinois University, DeKalb, IL 60115, United States of America; Tianjin University, CHINA

## Abstract

Detecting disease-related gene modules by analyzing gene expression data is of great significance. It is helpful for exploratory analysis of the interaction mechanisms of genes under complex disease phenotypes. The multi-label propagation algorithm (MLPA) has been widely used in module detection for its fast and easy implementation. The accuracy of MLPA greatly depends on the connections between nodes, and most existing research focuses on measuring the similarity between nodes. However, MLPA does not perform well with loose connections between disease-related genes. Moreover, the biological significance of modules obtained by MLPA has not been demonstrated. To solve these problems, we designed a double label propagation clustering algorithm (DLPCA) based on MLPA to study Huntington’s disease. In DLPCA, in addition to category labels, we introduced pathogenic labels to supervise the process of multi-label propagation clustering. The pathogenic labels contain pathogenic information about disease genes and the hierarchical structure of gene expression data. Experimental results demonstrated the superior performance of DLPCA compared with other conventional gene-clustering algorithms.

## Introduction

High throughput biotechnologies have been routinely used in biological and biomedical research. As a result, tremendous amounts of large-scale omics data have been generated, providing not only great opportunities but also challenges for understanding the molecular mechanisms of complex diseases [1]. Detecting disease-related gene modules by analyzing gene expression data represents one of these opportunities and challenges. Genes with similar expression patterns, as well as those with similar functions, are more likely to be regulated via the same mechanisms [2]. Therefore, we can extract disease-related molecular mechanisms through gene co-expression analysis if the genes involved in the mechanism form a significant co-expression gene module that contains known disease genes [3, 4]. The essence of such co-expression analysis is a clustering problem.

Gene expression data usually share characteristics such as small sample size, high dimensionality, and large amounts of noise. Generally, dimensionality reduction approaches and genome-wide biological network analysis methods have been widely studied for analyzing these data. To understand the interaction mechanisms of genes under complex disease phenotypes, biological network analysis is more appropriate [5]. A gene co-expression network (GCN) is usually constructed by measuring gene expression similarity, which represents the co-expression relationship between genes [6]. Each node in the network represents a single gene, and an edge connecting two genes indicates the co-expression [6].

Label propagation algorithms have been shown to be fast and easy to implement for analyzing large-scale complex networks [7]. Thus, these algorithms have been widely applied in text information retrieval [8, 9], multimedia annotation [10, 11], and community discovery [12–15]. A label propagation algorithm is a semi-supervised learning method based on a graph, which uses labels of some nodes to propagate and mark unlabeled nodes in the network [16]. If the number of label categories for one node exceeds two, the multi-label propagation algorithm (MLPA) is widely used. When the labels of nodes in the network are stable, nodes with the same label will be grouped into one specific category. MLPA is known to be fast and efficient for clustering [17]. The accuracy of MLPA depends heavily on the similarity measure between nodes. Most existing methods focus on developing better similarity measures to improve the performance of MLPA [18]. Cheng [19] measured the similarities between nodes with a sparsity induced similarity measure and conducted classification based on the label propagation results. Wang [20] studied label propagation between heterogeneous networks and proposed a strategy to propagate label information in a disorder-disease gene network. Tian [21] reconstructed a similarity matrix based on a weighted linear combination method. These methods improved the accuracy of MLPA from the similarity measure between nodes, though the biological significance of gene sets obtained by MLPA has not been demonstrated, and the hierarchical structures of gene expression data have not been fully used. In addition, the significance of a disease-related gene module performs poorly with loose connections between disease genes when using conventional gene clustering algorithms [22].

In the past few years, several network-based analysis methods to identify disease-related genes [23, 24] or disease-related microRNAs [25] have been proposed. A new local enrichment analysis method for disease-related genes identification has also been proposed [26]. These methods select the top genes of a ranking list as the most likely disease genes and have improved the accuracy of disease gene prediction. Disease-related genes are selected one by one by using these methods. Considering the complex characteristics of complex diseases [27, 28] and the fact that different molecules often work together to play their roles effectively, it is better to detect disease-related modules, which is helpful for understanding the modular mechanisms during disease progression.

Because biological experiments are time consuming, only a small amount of labeled data is present in biological databases. It is particularly urgent to develop efficient and effective computational methods that make full use of the label information for the small number of samples. Therefore, we developed a double label propagation clustering algorithm (DLPCA) for disease-related gene module detection. Compared with MLPA, DLPCA fully uses pathogenic information for sample genes and the hierarchical structure of biological networks while maintaining a fast running speed. In DLPCA, we used pathogenic labels, which represent pathogenic information for genes and the hierarchical structure of the gene co-expression network to supervise the process of category label propagation clustering. Because the DLPCA contains a semi-supervised pathogenic label propagation step, the clustering results have a clear biological meaning. Moreover, to accelerate convergence speed and improve the robustness of the clustering results, we also proposed a seed node selection method based on the local topological structure of a gene co-expression network. Experimental results demonstrated the feasibility and effectiveness of DLPCA as well as the superior performance of DLPCA compared with other conventional gene clustering algorithms.

The rest of this study is organized as follows: Materials used in our study and methods proposed in this paper are presented in Section 2. Experiments that analyze the performance of DLPCA and the overall discussion of DLPCA are reported in Section 3. Conclusions, along with some suggestions for future research, are presented in Section 4.

## Materials and methods

In this section, first, the gene expression data used in our study are described. Next, the construction of the gene co-expression network is briefly introduced. Then, we present the seed selection method based on local topological information. Finally, we describe the DLPCA.

### Gene expression data

The gene expression data used in our study were RNA-seq data downloaded from http://www.hdinhd.org. The data were obtained from the striatum tissue of 6-month-old Huntington’s disease (HD) mice. The gene expression data contain 4 genotypes, including polyQ 92, polyQ 111, polyQ 140, and polyQ 175. Each genotype has 8 replications. Thus, the gene expression data comprise 32 samples in total. The gene expression data contain 23,351 genes. After removal of genes with insignificant expression changes, 9578 genes remain for further consideration. The data on modifier genes were from Langfelder [29], which contain 520 genes in the training set, including 89 disease genes and 431 non-disease genes.

HD is a type of neurodegenerative diseases that is reported to be caused by a triplet repeat elongation in the Huntington gene (IT15), which leads to neuronal malfunction and degeneration through numerous interactions between genes and a number of different molecular pathways. The course of the disease is a constant progression of symptoms lasting 15 to 20 years after diagnosis and eventually leading to death. Several molecular mechanisms are involved in HD that lead to neuronal dysfunction. Genes with similar expression patterns are usually regulated via the same mechanism, forming modules in the gene co-expression network. Accordingly, if a module contains a relatively large number of disease genes, the biological function of the module may be highly relevant to the disease. This explains why we seek to extract disease-related modules from the gene co-expression network of HD.

### Construction of the gene co-expression network

To conduct the multi-label propagation algorithm, we must construct a gene co-expression network using gene expression data. The gene co-expression network used in our study was constructed using the WGCNA software package [30, 31]. As a scale-free network largely corresponds with biological networks, we used the WGCNA software package in our study to ensure that the gene co-expression network is scale-free [32]. Let *x*_*i*_ denote the expression profile of gene *i* and *x*_*j*_ denote the expression profile of gene *j*. The weight of the connection between gene *i* and gene *j* is *w*_*ij*_, where *w*_*ij*_ = |*cor*(*x*_*i*_, *x*_*j*_)|^*β*^. The parameter *β* is a soft threshold, which is set as the minimal positive integer that ensures the scale-free topology fit of the gene co-expression network is no more than 0.8. It should be noted that the stronger the Pearson correlation, the larger the weight [30, 31]. In the co-expression network *G* = (*V*, *E*), *V* is the set of nodes, where one node corresponds to a gene. *E* is the set of edges, showing the mutual interactions between genes. *W* = [*w*_*ij*_] is the weight matrix of the gene co-expression network. The adjacency matrix is *A* = [*a*_*ij*_], where *a*_*ij*_ represents the interactions between node *i* and *j*. The calculation of *a*_*ij*_ is given by

$$a_{ij}=\left\{\begin{array}{cc} 1, & \text{if}\;\;\;w_{ij}\neq 0,\\ 0, & \text{else}. \end{array}\right.$$ (1)

The transition probability matrix is *P* = [*p*_*ij*_], where *p*_*ij*_ denotes the probability of transition from node *i* to node *j*. In fact, *P* is a normalized matrix of *W* along the row vector. The calculation of *p*_*ij*_ is given by

$$p_{ij}=\left\{\begin{array}{cc} \frac{w_{ij}}{\sum_{j\in N_{i}}w_{ij}}, & \text{if}\;\;N_{i}\neq \emptyset ,\\ 0, & \text{else}, \end{array}\right.$$ (2)

where *N*_*i*_ is the set of neighboring nodes of node *i* in the gene co-expression network.

### Selection of seed nodes

Gene co-expression networks have been shown to exhibit a modular structure. Good seed nodes are helpful for module detection [33]. According to the local topological structure of the gene co-expression network, we selected seed nodes to accelerate the convergence speed and improve the cluster robustness of MLPA [34]. Since nodes with large clustering coefficients and large degrees can spread information quickly and easily, we selected seed nodes based on degree and clustering coefficient. The details for seed nodes selection are shown below.

**Step 1.** Compute the clustering coefficient of node *i*, $c_{i}=\frac{2\sum_{j,k\in N_{i}}a_{jk}}{d_{i}\cdot \left(d_{i}-1\right)}$, where *d*_*i*_ represents the degree of node *i*. Then, rank all the nodes in descending order according to the clustering coefficient *c*_*i*_. *R*_*c*_*i*__ represents the ranking of node *i* in the ranked list.
**Step 2.** Compute the degree of node *i*, *d*_*i*_ = ∑_*j*∈*N*_*i*__ *a*_*ij*_. Then, rank all the nodes in descending order according to their degrees. *R*_*d*_*i*__ represents the ranking of node *i* in the rank list.
**Step 3.** The rank-product strategy [35] yields the comprehensive ranking of node *i*, $R_{i}={\left(R_{c_{i}}\cdot R_{d_{i}}\right)}^{\frac{1}{2}}$.
**Step 4.** Rank *R*_*i*_, *i* ∈ *V*, in ascending order and select the first *m* nodes as seeds. We denote the seed set as *S*, while the category label of seed node *i* is *f*_*i*_, *i* ∈ *S*.

It should be clarified that the category labels of seeds are used to extract modules from the gene co-expression network. In the MLPA results nodes with the same category label are considered a module.

### Double label propagation clustering algorithm

To make full use of some genes with pathogenic information and improve the biological meaning of the clusters, we take the pathogenic information of genes into consideration during category label propagation. The initial pathogenic label of a gene is given by Eq (3).

$$l_{i}^{0}=\left\{\begin{array}{cc} 1, & \text{if}\;\;i\;\;\text{is}\;\;a\;\;\text{disease}\;\;\text{gene},\\ -1, & \text{if}\;\;i\;\;\text{is}\;\;a\;\;\text{non}-\text{disease}\;\;\text{gene},\\ 0, & \text{otherwise}. \end{array}\right.$$ (3)

We conduct semi-supervised pathogenic label propagation using the known pathogenic information of some genes to supervise the multi-label propagation clustering, thus obtaining the most likely disease-related modules.

**Definition 1 Category label update rule.** When multi-label propagation is used to detect functional modules, the following update rule for the category labels is used during the label propagation.

The category label of node *i* is

$$f\left(i\right)=\underset{f_{n}}{\mathrm{argmax}}\left(\lambda_{1}\sum_{j\in N_{i}^{f_{n}}}w_{ij}+\lambda_{2}\sum_{j\in N_{i}^{f_{n}}}a_{ij}+\lambda_{3}\sum_{j\in N_{i}^{f_{n}}}l_{j}^{t-1}\cdot l_{i}^{t-1}\right)$$ (4)

where $N_{i}^{f_{n}}$ represents the neighboring nodes of node *i* with the category label *f*_*n*_, *n* ∈ *S*. *λ*_1_, *λ*_2_, *λ*_3_ are parameters. *λ*_1_ controls the effects caused by weighted connectivity. *λ*_2_ controls the effects caused by the number of neighboring nodes. *λ*_3_ controls the effects caused by the pathogenic information of the neighboring nodes. We assumed that the weighted connectivity, the degree and the pathogenic information have equal influence on the category label of a gene.

**Definition 2 Pathogenic label update rule.** Based on the topological structure of the gene co-expression network, update the pathogenic label of other nodes in the network by using the small amounts of genes with known pathogenic information.

The pathogenic label of node *i* is

$$l_{i}^{t}=\beta_{1}\sum_{j\in N_{i}^{f(i)}}p_{ij}l_{j}^{t-1}+\beta_{2}\sum_{j\in N_{i}^{-f(i)}}p_{ij}l_{j}^{t-1}+\beta_{3}l_{i}^{t-1}$$ (5)

where $l_{i}^{t}$ is the pathogenic label of node *i* at the *t*th iteration, $N_{i}^{f(i)}$ represents the neighboring nodes of node *i* whose category label is the same as node *i*. $N_{i}^{-f(i)}$ represents the neighboring nodes of node *i* whose category label is different from node *i*. The symbols *β*_1_, *β*_2_, *β*_3_ are parameters. The parameter *β*_1_ regulates the pathogenic effects caused by the nodes in $N_{i}^{f(i)}$. The parameter *β*_2_ regulates the pathogenic effects caused by the nodes in $N_{i}^{-f(i)}$. The $\beta_{3}l_{i}^{t-1}$ ensures that the pathogenic label of node *i* is stable during the pathogenic label updating process. In addition, *β*_1_ + *β*_2_ + *β*_3_ = 1 ensures that the pathogenic label is ultimately convergent [20].

**Definition 3 Conditions for termination of iteration.** The conditions for termination of DLPCA are that the category labels of the nodes in the network stop changing or that the change of the pathogenic information for any node is less than the threshold. In this study, the threshold is 0.1.

The DLPCA procedure is summarized in Algorithm 1.

Algorithm 1: DLPCA

**Input:** gene expression data, parameters: *λ*_1_, *λ*_2_, *λ*_3_, *β*_1_, *β*_2_, *β*_3_

**Input:** pathogenic labels of some genes

**Output:** gene category label

1: Construct the gene co-expression network. Compute the weight matrix, the adjacency matrix, and the transition probability matrix

2: Select the seed nodes

3: **repeat**

4:   Update the gene category label according to Eq (4)

5:   Update the gene pathogenic label according to Eq (5)

6: **until** conditions for termination are satisfied

7: **return** gene category label

## Results and discussion

In this section, the selection of parameters is first described. Next, we compare the DLPCA with other conventional methods to demonstrate the superior performance of DLPCA. Second, we analyze the time complexity of DLPCA and MLPA. Third, we conduct an enrichment analysis of the modules obtained using DLPCA. Finally, we present an overall discussion to clearly illustrate the purpose of this study and demonstrate the key point of the algorithm.

### Parameter selection

**Fig 1:**
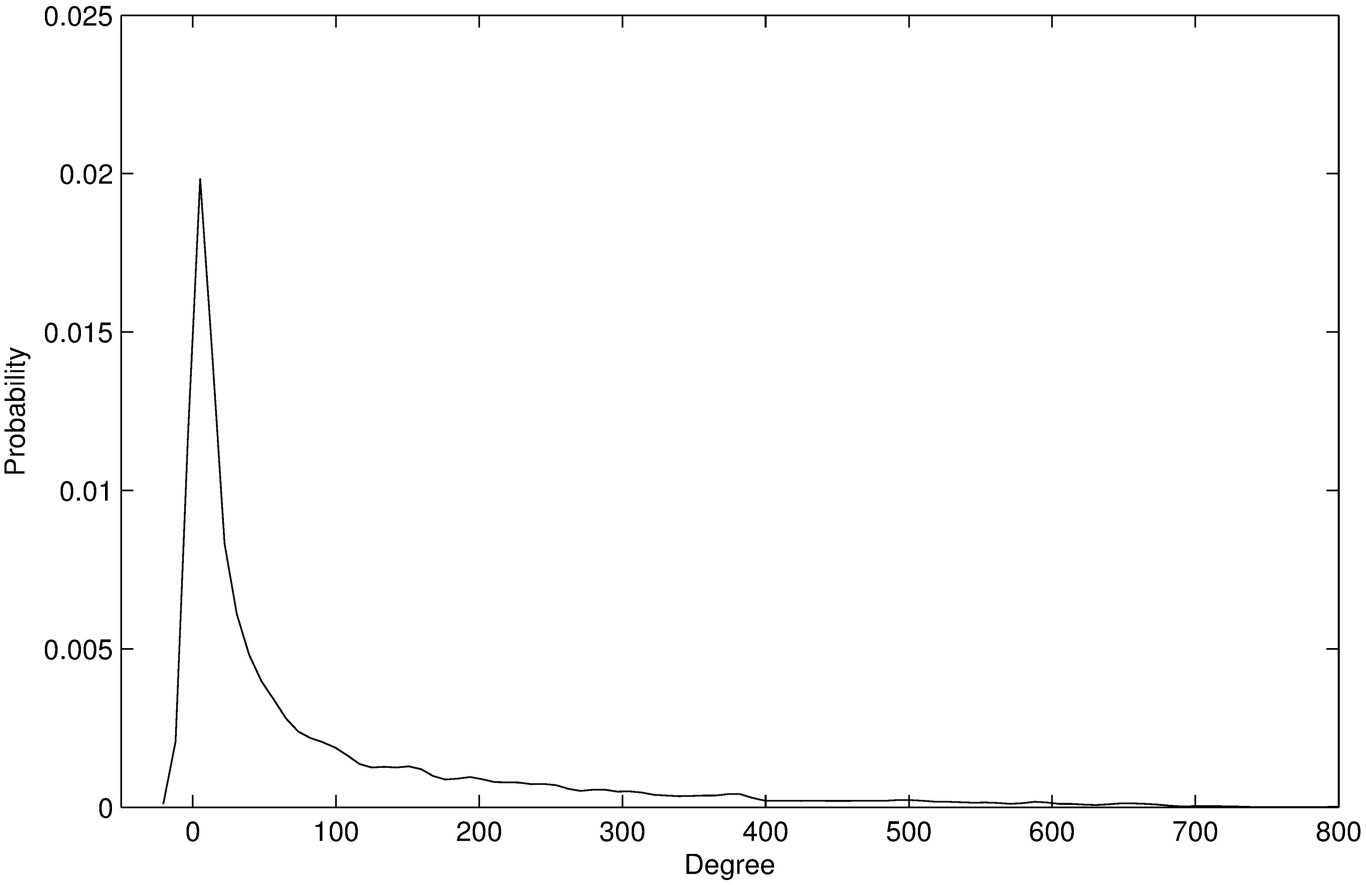
The probability density distribution of weighted connectivity in the co-expression network. The probability density distribution obeys a power-law distribution, showing the biological reasonability of the co-expression network.

**Fig 2:**
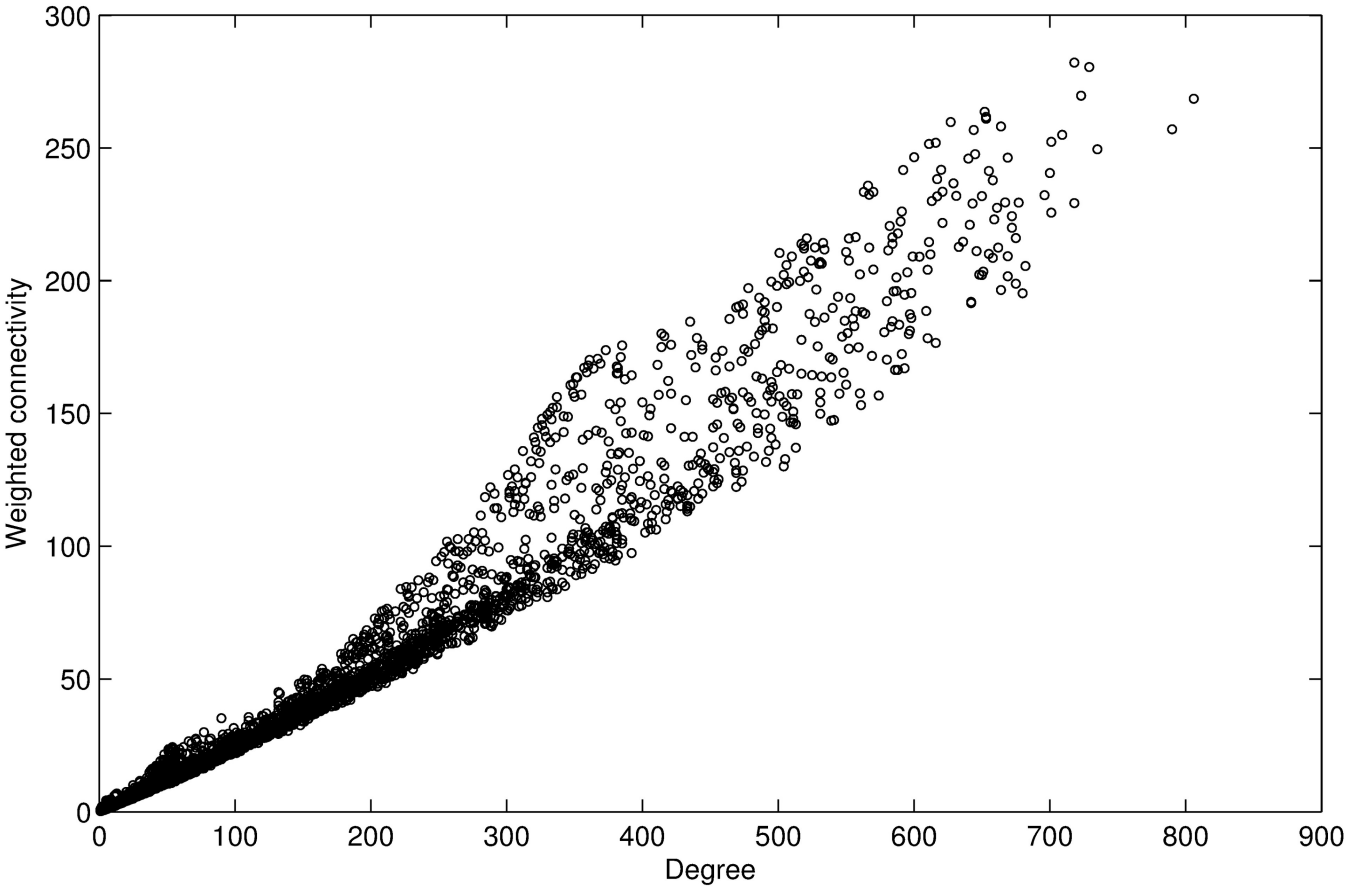
The relationship between degree and weighted connectivity. The scatterplot shows a near-linear correlation between the degree and the weighted connectivity.

Topological information of the co-expression network is shown in Table 1. Fig 1 indicates the biological reasonability of the gene co-expression network. As shown in Fig 2, the degree and the weighted connectivity exhibit a near-linear correlation. The scatter represents the isolated node in the gene co-expression network.

**Table 1:** Topological information of the gene co-expression network.

Node number	9587
Average weight	0.291
Average weighted connectivity	18.97
Average degree	65.23
Scatters	536

According to Table 1 (the average degree and the average weighted connectivity of the co-expression network) and Fig 2 (the near-linear correlation between the degree and the weighted connectivity), we know that the correlation coefficient is roughly equal to the ratio of the average degree to the average weighted connectivity. Considering that the weighted connectivity, degree, and pathogenic information have equal influence on the category label of a node in the present study, we obtained *λ*_1_: *λ*_2_ = 65.23: 18.97. Following the semi-supervised pathogenic label propagation, the average pathogenic information of all nodes is 0.236. We then obtained *λ*_2_: *λ*_3_ ≈ 1: 0.236 × 0.236. Therefore, we set *λ*_1_ = 3.44, *λ*_2_ = 1.0, *λ*_3_ = 20.0 for computational convenience. It should be noted that traditional MLPA only considers weighted connectivity in category label propagation.

For pathogenic label updating, different parameter combinations may yield different clustering results. To analyze the impact of different parameter combinations on the clustering results, we defined two groups of parameters. Group I is *β*_1_ = *β*_2_ = 0.15, *β*_3_ = 0.7. Group II is *β*_1_ = 0.2, *β*_2_ = 0.1, *β*_3_ = 0.7.

In addition, to analyze the impact of parameter *m* on the clustering results, we selected 350, 500, and 750 seed nodes to conduct category label propagation for the traditional MLPA method and the DLPCA method, respectively.

### Performance comparison between DLPCA and MLPA

To evaluate the performance of different clustering algorithms, the following criteria were used: the coverage, the significance of the disease-related module, and the significance of scatters. The coverage is defined as the ratio of genes in modules to all genes in the network. The significance of the disease-related module is defined as the ratio of disease genes to genes in the training set included in the module. The significance of scatters is defined as the ratio of disease genes to genes in the training set included in the scatters. The clustering results are improved along with increased significance of disease-related modules and decreased significance of scatters. The clustering results of MLPA and DLPCA are shown in Table 2.

**Table 2:** The clustering results of each experiment.

Method	Seed num	Module num	Coverage	Avg module size	Avg module significance	Scatter significance	Disease modules		
							num	Avg size	Avg significance
DLPCA *β*_1_ = *β*_2_	350	30	0.750	239.7	0.4240	0.1067	9	539.2	0.8952
	500	31	0.796	246.2	0.4492	0.1218	10	412.5	0.8085
	650	40	0.750	179.8	0.3518	0.1053	10	595.7	0.8090
DLPCA *β*_1_ > *β*_2_	350	25	0.749	276.3	0.1825	0.1491	6	608.8	0.3949
	500	30	0.750	239.7	0.2294	0.1447	8	787.8	0.4015
	650	37	0.752	194.8	0.1595	0.1389	7	710.7	0.4191
MLPA	350	39	0.723	177.8	0.1279	0.1791	6	841.9	0.2923
	500	41	0.724	169.3	0.1465	0.2246	8	839.8	0.3113
	650	116	0.655	80.0	0.1757	0.1803	13	390.5	0.4001

**Fig 3:**
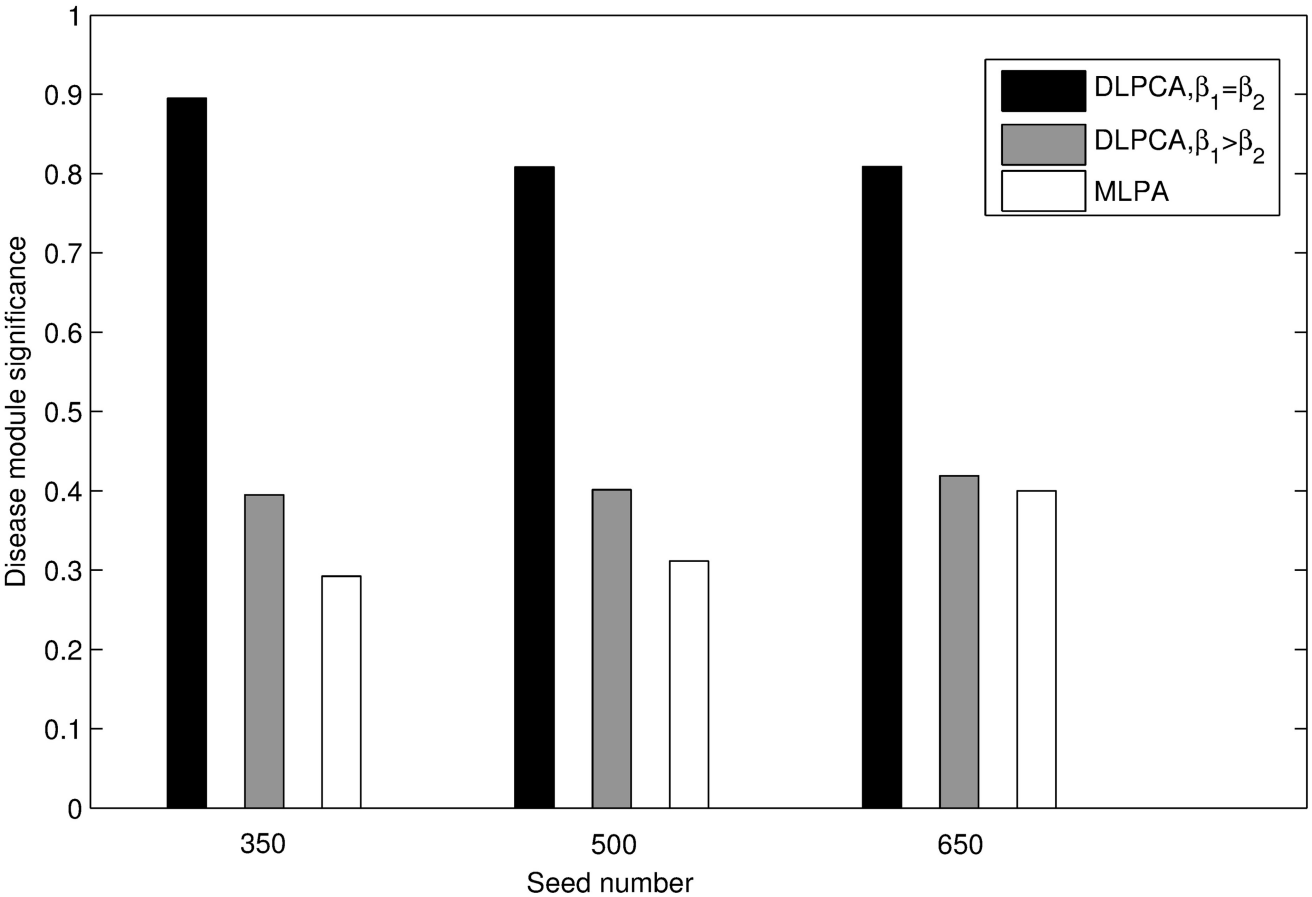
Comparison of the average significance of disease-related modules obtained by DLPCA with *β*_1_ = *β*_2_, DLPCA with *β*_1_ > *β*_2_, and MLPCA. Each grouped bar chart represents the results of different approaches with the same numbers of seeds.

**Fig 4:**
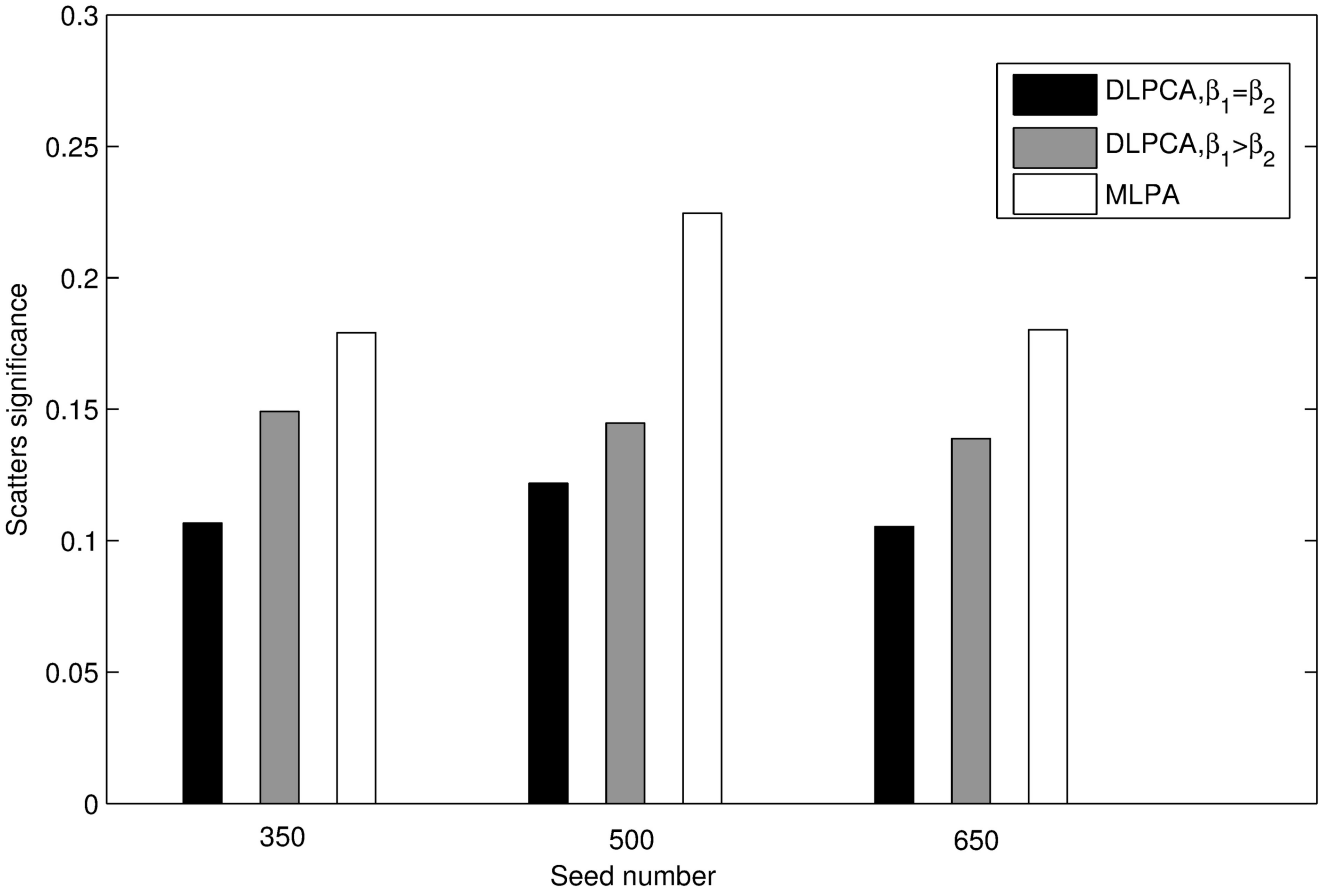
Comparison of the significance of scatters obtained by DLPCA with *β*_1_ = *β*_2_, DLPCA with *β*_1_ > *β*_2_, and MLPCA. Each grouped bar chart represents the results of different approaches with the same numbers of seeds.

Figs 3 and 4 present the clustering results of these methods. As illustrated in Fig 3, with the same seed numbers, the average significance of disease-related modules obtained using DLPCA with *β*_1_ = *β*_2_ (the average significances of disease modules is 0.837) is higher than that of the other experiments. As shown in Fig 4, with the same number of seeds, the significance of scatters obtained using DLPCA with *β*_1_ = *β*_2_ (the average significances of scatters is 0.111) is lower than that of the other experiments. It is also clear that the average significance of disease-related modules with different numbers of seeds obtained using DLPCA are similar (Fig 3). The significance of scatters obtained using different numbers of seeds in DLPCA are also similar (Fig 4). These results suggest that the clustering results of DLPCA are insensitive to seed number.

Figs 3 and 4 also show that the clustering results of DLPCA with *β*_1_ = *β*_2_ are much better than that of DLPCA with *β*_1_ > *β*_2_, indicating that different parameter combinations significantly impact the clustering results. When the coefficients of the two category labels are equal, i.e., *β*_1_ = *β*_2_ in DLPCA, the average significance of the disease-related modules (the average significance of the disease modules is 0.837) and the significance of the scatters (the significances of the scatters is 0.111) are the best. It demonstrates that DLPCA with *β*_1_ = *β*_2_ can separate disease genes from non-disease genes very well during the clustering process. When the coefficients of the two category labels are not equal, generally, the neighboring nodes whose category labels are the same as that of node *i* have a greater impact on the pathogenic label of node *i*, i.e., *β*_1_ > *β*_2_ in DLPCA. Affected by the interaction of the category label and the pathogenic label, DLPCA with *β*_1_ > *β*_2_ may easily fall into local optimization. This situation could be prevented by setting *β*_1_ = *β*_2_, ensuring that category label updating is immune to pathogenic label updating.

Furthermore, clusters obtained by MLPA have often been shown to contain few genes, which is also confirmed in this study (the average module size of MLPA is shown in Table 2). The experimental results also suggest that MLPA fails to effectively separate disease genes from non-disease genes. However, DLPCA contains a semi-supervised pathogenic label propagation step, which is very helpful for separating disease genes from non-disease genes. DLPCA greatly improves the average significance of disease-related modules compared with MLPA. In the DLPCA results, the sizes of disease-related modules are between 20 and 300 except for two large modules whose sizes are larger than 1000. In summary, DLPCA can effectively improve the performance of clustering results by selecting the appropriate parameters as suggested in our study.

### Performance comparison between DLPCA and DCOTCA

To compare the performance of DLPCA with other algorithms, we conducted experiments using the dynamic cut-off tree clustering algorithm (DCOTCA). The clustering results are illustrated in Table 3.

**Table 3:** The clustering results of dynamic cut-off clustering tree algorithm.

DCOTCA	Module num	Coverage	Avg module size	Avg module significance	Scatter significance	Disease modules		
						num	avg size	avg significance
Experiment1	18	0.560	297.4	0.1613	0.1462	14	334.2	0.2064
Experiment2	22	0.568	247.4	0.1572	0.1473	16	286.3	0.2161
Experiment3	34	0.565	159.2	0.1454	0.1521	19	187.6	0.2603
Experiment4	49	0.603	117.9	0.1672	0.1534	26	133.3	0.3030
Experiment5	87	0.606	66.8	0.1950	0.1518	32	82.2	0.3891

**Fig 5:**
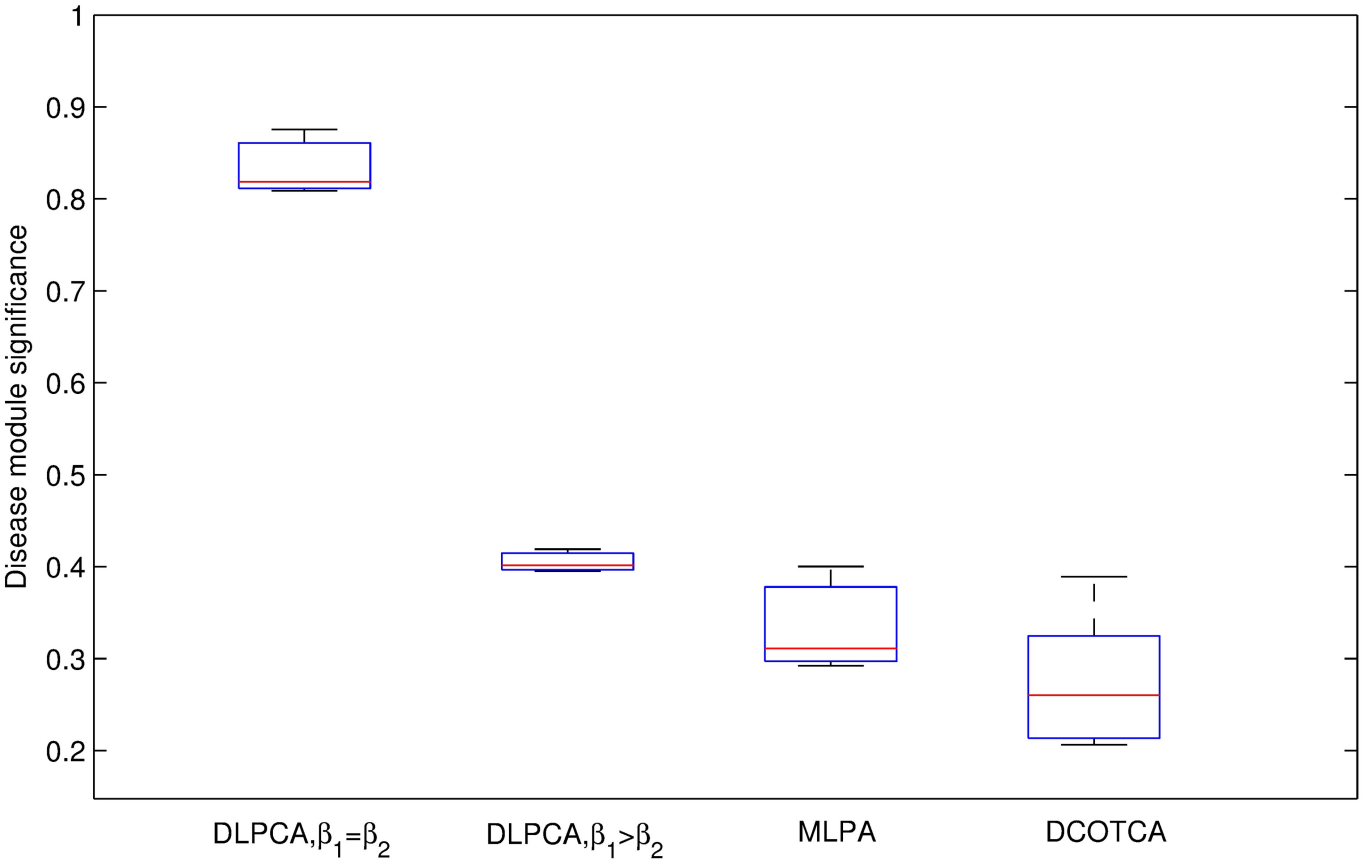
Comparison of the average significance of disease-related modules obtained by DLPCA with *β*_1_ = *β*_2_, DLPCA with *β*_1_ > *β*_2_, and MLPCA. Each box shows the average significance of disease-related modules using an approach with different numbers of seeds.

**Fig 6:**
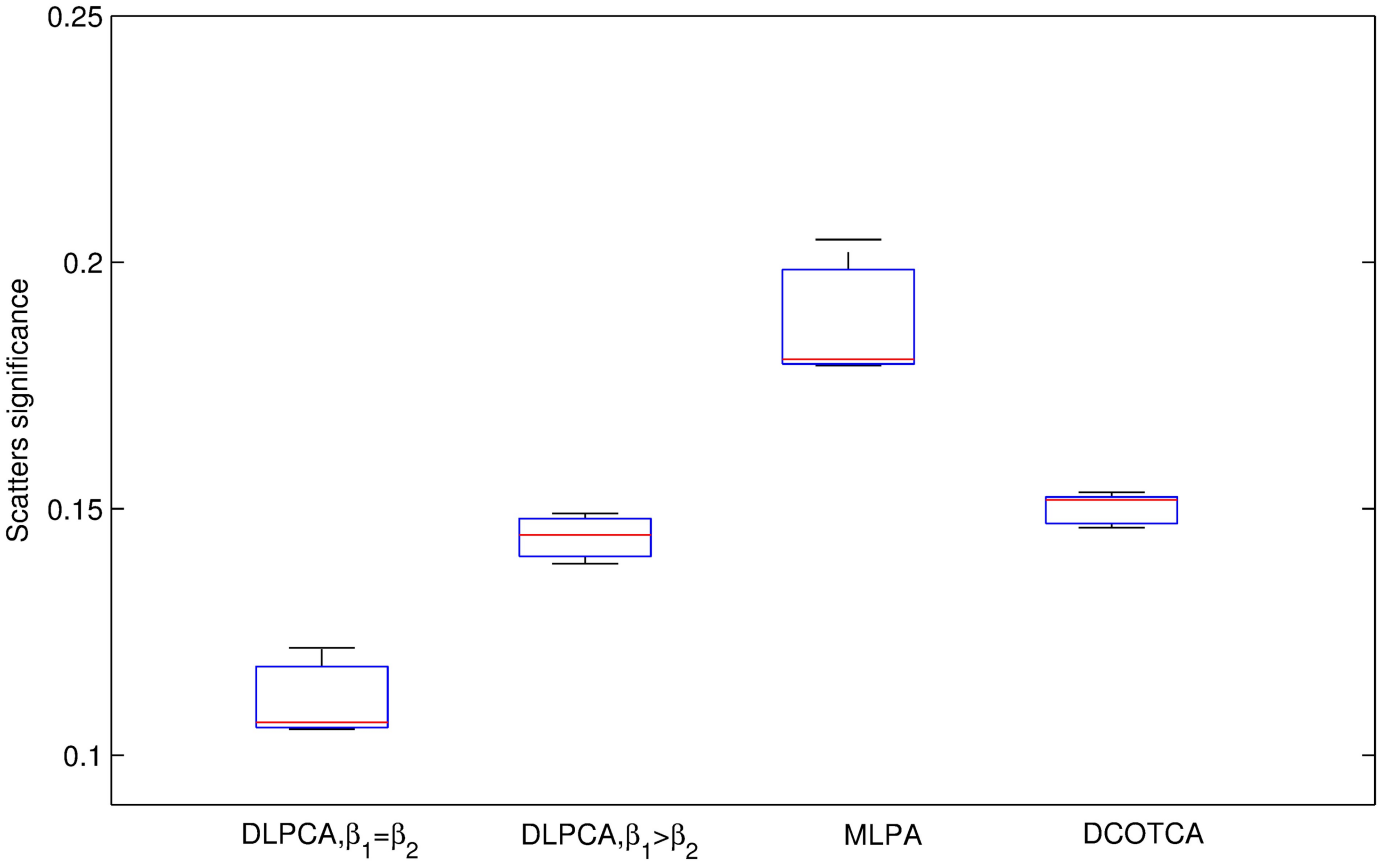
Comparison of the significance for scatters obtained by DLPCA with *β*_1_ = *β*_2_, DLPCA with *β*_1_ > *β*_2_, and MLPCA. Each box shows the significance of scatters using an approach with different numbers of seeds.

**Fig 7:**
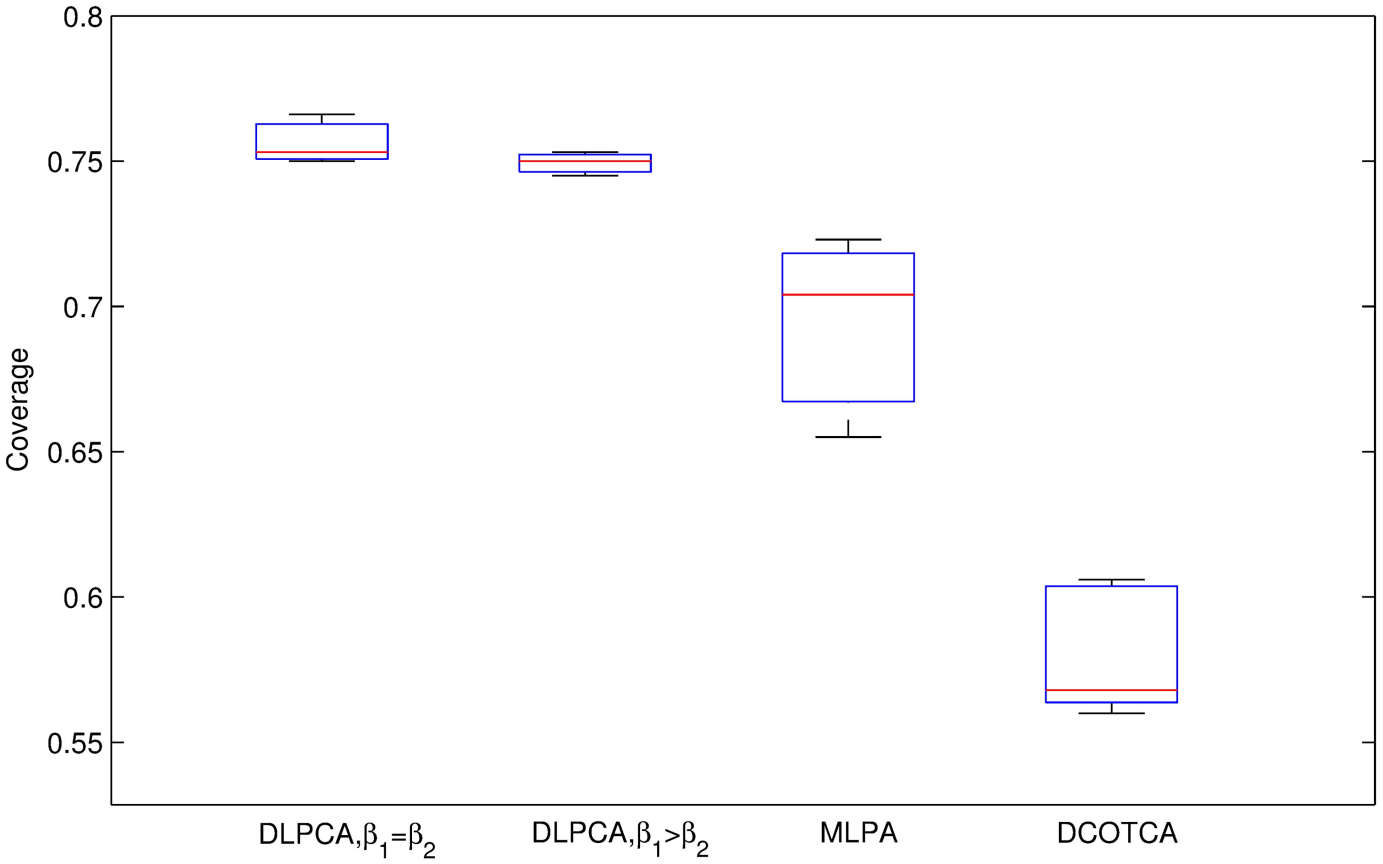
Comparison of the coverage of DLPCA with *β*_1_ = *β*_2_, DLPCA with *β*_1_ > *β*_2_, and MLPCA. Each box shows the coverage using an approach with different numbers of seeds.

Fig 5 shows that the average significance of disease-related modules using DLPCA (0.837) is much higher than that of DCOTCA (0.275). Fig 6 shows that the average significance of scatters using DLPCA (0.111) is lower than that of DCOTCA (0.150). From Fig 7, we can see that DLPCA also provides much better coverage than other experiments. To summarize, the clustering results of DLPCA are better than those of DCOTCA (Figs 5, 6 and 7).

### Time complexity analysis of DLPCA and MLPA

When the number of nodes in the gene co-expression network is *n* and the number of seed nodes is *m*, the time complexity of MLPA in each iteration is *O*(*m* ⋅ *n*^2^). Approximately 10 iterations in each MLPA experiment are needed to reach convergence (Table 4). Given the interaction of the category label and pathogenic label in DLPCA with *β*_1_ > *β*_2_, fewer iteration times are needed relative to DLPCA with *β*_1_ = *β*_2_ to reach convergence. During the category label propagation process, traditional MLPA only considers the impact of weighted connectivity on category label according to a static network; thus, the iteration process of MLPA is the fastest.

**Table 4:** The iteration times in each experiment.

Method	DLPCA, *β*_1_ = *β*_2_			DLPCA, *β*_1_ > *β*_2_			MLPA		
Seed num	350	500	650	350	500	650	350	500	650
Iteration times	12	10	14	8	10	11	7	11	8

The time of per iteration varies. Generally, increasing the seed number increases the time. The average time of each iteration is displayed in Table 5. Note that we used a server with the Linux operating system, 100 GB memory, and an Intel (R) Xeon (R) E5-2603 v3 @1.60GHZ CPU for the data analysis. The algorithm was run on Java 1.7.0_17.

**Table 5:** The average time per iteration in each experiment.

Method	DLPCA, *β*_1_ = *β*_2_			DLPCA, *β*_1_ > *β*_2_			MLPA		
Seed num	350	500	650	350	500	650	350	500	650
Time (hours)	3.1	5.8	6.3	2.9	4.9	6.4	2.6	3.9	5.6

### Enrichment analysis

In addition, we conducted enrichment analysis using the DAVID [36] to determine the biological function of the modules obtained using the DLPCA. The clustering results of DLPCA with *β*_1_ = *β*_2_ and 350 seed genes were used in the enrichment analysis. We listed annotation clusters with high enrichment scores (ES) for the 9 disease-related modules. We also investigated the enrichment annotations for another two modules that are not associated with the disease to analyze the factors that are not effected by or do not contribute to the disease. The detailed annotations of these modules are shown in Table 6.

**Table 6:** Functional annotation clustering for the modules obtained using DLPCA with *β*_1_ = *β*_2_ and 350 seed nodes.

DLPCA	Module size	Disease genes num	Non-disease genes num	Module sig	Functional annotation clustering	
					Annotation cluster	ES
Module1	91	19	1	0.95	Metal-binding	2.69
					Sequence repeat	2.54
					Calcium icon binding	2.42
					Ribosome	2.14
Module2	109	12	0	1.0	Cytoskeleton	4.05
					Cell junction	3.67
					Calcium icon transport	2.57
					Oxytocin signaling pathway	2.04
Module3	81	9	0	1.0	Golgi apparatus	1.99
Module4	28	2	0	1.0	Ubl conjugation pathway	2.12
Module5	31	2	0	1.0	Nucleotide-binding	3.67
Module6	104	3	0	1.0	Postsynaptic density	3.07
					Endoplasmic reticulum	2.04
Module7	71	5	0	1.0	Retrograde endocannabinoid signaling	3.67
					Membrane	3.01
Module8	1907	18	1	0.95	Nucleotide-binding	9.75
					Chaperone	6.58
					DnaJ domain	6.04
					F-box domain	5.69
					Microtubule	4.94
Module9	2431	15	79	0.16	Zinc, metal-binding	41.41
					Protein transport	10.1
					Ligase	9.87
					Transcription regulation	9.52
					Zinc figure	9.13
Module10	623	0	137	–	Lysosome	8.04
					Cilium	7.36
					Glycoprotein	5.95
					Extracellular matrix	5.48
Module11	1524	0	256	–	Synapse	28.21
					Ion transport	10.12
					Glycoprotein	6.83
					Fatty acid	5.74

For module 1 (disease-related module with 91 genes, including 19 disease genes and 1 non-disease genes), identified annotation clusters include metal-binding (cluster 1 with enrichment score 2.69), sequence repeat (cluster 2 with enrichment score 2.54), calcium icon binding (cluster 3 with enrichment score 2.42) and ribosome (cluster 4 with enrichment score 2.14). The significance of the module is 0.95. The annotations for the module suggest that HD maybe associated with these functional annotations above. In fact, HD is caused by the excessive repetition of CAG in the fourth chromosome, which corresponds to the functional annotation, i.e., sequence repeat, of the disease-related module. On the other hand, for module 10 (the non-disease-related module with 623 genes, including 0 disease genes and 137 non-disease genes), annotation clusters such as lysosome (cluster 1 with enrichment score 8.04), cilium (cluster 2 with enrichment score 7.36), Glycoprotein (cluster 3 with enrichment score 5.95) and extracellular matrix (cluster 4 with enrichment score 5.48) were identified. Since the module contains no disease genes, the above functions are most likely not affected by the disease.

The pathology of Huntington disease is very complex and many factors are involved in the disease progression, including inflammation, impaired metabolic pathways, protein mis-folding [37–39], etc. The enrichment analysis results demonstrate that a disease-related module often contains many functional annotations that could reflect complicated pathologies and also verifies the effectiveness and reasonability of the DLPCA.

### An overall discussion

Although tremendous amounts of omics data are being collected along with the rapid development of high-throughput technology, only a small amount of data contain clearly biological annotations, e.g., pathogenic information on genes for specific complex diseases. The challenge is how to fully utilize the small amounts of labeled data to discover effective knowledge from the genome-wide data.

**Fig 8:**
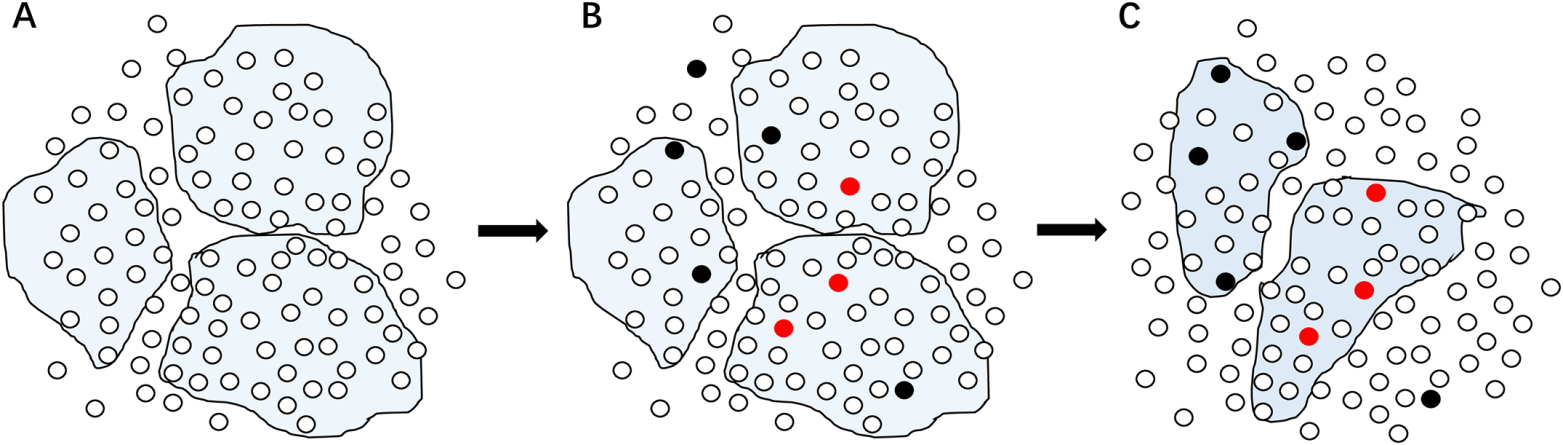
Illustration of DLPCA compared with MLPA. (A) The modules in the gene co-expression network obtained using MLPA. (B) Introduction of the pathogenic information of some genes. Here, red nodes represent disease genes and black nodes represent non-disease genes. (C) The new modular structures in the gene co-expression network obtained using DLPCA.

The DLPCA designed in this study aims to mine the most likely disease-related modules from gene expression data by making full use of the pathogenic information of a small number of genes. In addition, DLPCA also makes full use of the hierarchical structures in the network, including the structures represented by the category labels and those represented by the pathogenic labels. This computational method can improve the efficiency and effectiveness of downstream biological experimental analysis. To clarify the main idea of this study and the key point of the DLPCA, we have drawn Fig 8 to clearly demonstrate the properties of DLPCA compared with MLPA. DLPCA is helpful for classifying genes with similar biological properties into one module. Compared with MLPA, DLPCA effectively improves the biological significance of the gene clusters.

## Conclusions

In this study, we designed a double label propagation clustering algorithm for detecting disease-related modules. This algorithm takes the pathogenic information of genes as a property of nodes in the gene co-expression network. During the clustering process of MLPA, DLPCA not only considers the topological structures of the network but also the biological properties of the nodes in the network. In addition, to accelerate convergence and improve cluster robustness, we also proposed a seed selection strategy according to the local topological structure of the gene co-expression network. Compared with the aforementioned conventional methods, DLPCA effectively improves the accuracy of disease-related module identification. However, it should be stated that DLPCA could be applied equally well to other biological networks and genomic data.

Recently, new module detection methods integrating different network structures have been proposed [40]. Generally, the accuracy of disease module detection may be further improved by integrating other biological data as well as gene expression data, especially for gene expression data characterized by large amounts of noise. Therefore, our future efforts will focus on integrating multi-source biological data to further improve the accuracy of disease-related modules.
